# Yamasachi Wine: Characterization of Aroma Compounds and Its Masking Effect Against Phenolic Off-Flavor

**DOI:** 10.3390/foods15183195

**Published:** 2026-09-09

**Authors:** Noriko Minami, Akihiro Yamaguchi, Masafumi Sakurai, Fumie Kabashima, Masahiro Horiuchi, Satoru Nakagawa, Teruo Sone

**Affiliations:** 1 Graduate School of Global Food Resources, Hokkaido University, Kita 9, Nishi 9, Kita-ku, Sapporo 060-0809, Hokkaido, Japan; minamin@jfrl.or.jp; 2 Japan Food Research Laboratories, 2-3 Bunkyo, Chitose 066-0052, Hokkaido, Japan; 3 Department of Food Science and Human Wellness, Rakuno Gakuen University, Midorimachi 582, Bunkyodai, Ebetsu 069-8501, Hokkaido, Japan; ymg-akh@rakuno.ac.jp; 4 LECO Japan, Sumitomo Fudosan Shiba Building No. 4, 2-13-4 Shiba, Minato-ku, Tokyo 105-0014, Japan; masafumi_sakurai@leco.com (M.S.); fumie_kabashima@leco.com (F.K.); 5 Takata Koryo Co., Ltd., 7-22-2 Tsukaguchihonmachi, Amagasaki 661-0001, Hyogo, Japan; m.horiuchi@takatakoryo.co.jp; 6 Research Faculty of Media and Communication, Hokkaido University, Kita 17 Nishi 8, Kita-ku, Sapporo 060-0817, Hokkaido, Japan; nakagawa@imc.hokudai.ac.jp; 7 Research Faculty of Agriculture, Hokkaido University, Kita 9, Nishi 9, Kita-ku, Sapporo 060-0809, Hokkaido, Japan

**Keywords:** GC–TOFMS, multivariate statistical analysis, sensory analysis, *Vitis amurensis*

## Abstract

Yamasachi, an original red wine grape in Hokkaido, is a hybrid variety between ‘Seibel 13053’ and an indigenous wild grape. This study aimed to elucidate the aromatic compounds characterizing Yamasachi wine. Two methods, instrumental analysis using gas chromatography combined with time-of-flight mass spectrometry (GC–TOFMS) and sensory perception, were employed. Seven unique key aroma compounds, linalool ethyl ether, vitispirane, Bois de Rose oxide, *trans*-2-hexen-1-ol, *cis*-3-hexen-1-ol, 4-ethylguaiacol, and 4-ethylphenol, were identified. Additionally, principal component analysis revealed strong perceptions of green pepper, horseradish, earthy, mushroom, black fruits, herbs, and spices in the Yamasachi wine aroma profile. Surprisingly, sensory analysis of Yamasachi wine did not detect any negative phenolic off-flavors; nevertheless, the higher 4-ethylguaiacol and 4-ethylphenol concentrations were detected. Spike experiments with these compounds repressed the unpleasant phenolic aroma in Yamasachi wine. These results suggest that Yamasachi wine has a masking effect on phenolic off-flavors.

## 1. Introduction

Recently, the number of wineries in Hokkaido, the northernmost prefecture of Japan, has rapidly increased, now exceeding 70. One of the reasons is global warming, which makes the climate more suitable for the cultivation of European wine grapes such as Pinot Noir and Chardonnay, attracting new winegrowers. Another reason is the expansion of the cultivation of cold-resistant grape varieties in the middle-east area in the island, where the vines face less than −20 °C temperature. Yamasachi is an original wine grape variety from Hokkaido bred in Ikeda town [1]. This is a hybrid cultivar of Kiyomi, a clone of Seibel 13053, selected to adapt to the cold climate, and a species of *Vitis amurensis* with excellent cold and freeze resistance [2]. Therefore, the hybrid cultivar acquired strong cold-hardiness, which reduces the need for measures to prevent wilting during winter months [3]. Yamasachi is the third international Japanese wine grape variety registered with the International Grape and Wine Organization (OIV) in 2020. The acreage of Yamasachi reached 34.7 ha in Hokkaido in 2021, as the third wide-grown red wine variety (Ministry of agriculture, forestry and fisheries: The 98th Statistical yearbook of crops, fruits and Nuts). Although official statistics on the annual production volume of Yamasachi wine are not publicly available, governmental reports indicate that the wine industry in Hokkaido has expanded substantially, reaching 76 wineries as of 2026. These factors suggest that, despite its limited production scale, Yamasachi represents a culturally and commercially relevant variety within the rapidly developing wine sector in Hokkaido. In addition to its regional importance, Yamasachi exhibits a sensory profile that differs markedly from European cultivars commonly grown in Hokkaido. However, the chemical basis of these distinctive sensory attributes remains unknown. Establishing the key aroma compounds of Yamasachi is essential for understanding the varietal characteristics, improving winemaking practices, and supporting quality control and branding of wines produced from this unique Japanese grape variety [4].

Aroma and flavor are the most important factors affecting wine quality and greatly influence consumer preferences and judgments [5]. Several studies have focused on the characteristic aroma of wines made of European grape varieties. More than 1000 volatile components, including esters, higher alcohols, terpenes, ketones, aldehydes, acids, lactones, C13 norisoprenoids, volatile phenols, thiols, and lactones play a role in the complex sensory characteristics of wine [6]. Each volatile compound contributes to an aroma, such as citrus, sweet, woody, flowery, honey, coconut, tropical, berry fruit, fermented, toasted, spicy, fusel alcohol, vanilla, and herbaceous [7,8], and some compounds are responsible for off-flavors in wine. For example, phenolic compounds, 4-ethylguaiacol and 4-ethylphenol contribute to the ‘barnyard’ or ‘medicinal’ odors [9].

Wines produced from Yamasachi have a distinct aroma compared to those produced from European grape varieties, such as Pinot Noir, Zweigelt, and Merlot, which are also produced in Hokkaido and are earthy and greenish. However, no studies have identified the compounds responsible for the characteristic aroma of Yamasachi wine. Untargeted analysis with gas chromatography combined with time-of-flight mass spectrometry (GC–TOFMS) is commonly used to separate and detect aromatic compounds such as phenols, alcohols, acids, norisoprenoids, esters, aldehydes, and terpenoids in wine samples [10]. The combined data from GC-TOFMS and sensory analysis can be examined using principal component analysis (PCA) to identify key aromas for the characterization of significant aroma compounds and sensory attributes [11].

This study aimed to establish the aroma profile of Yamasachi wine. Utilizing untargeted analysis of aroma compounds and sensory analysis, aroma compounds specifically present in Yamasachi wine were identified by comparison with wines made from Zweigelt and Pinot Noir, the major red grape varieties in Hokkaido. The aroma of wine is determined by the complex interactions among hundreds of volatile compounds, which can be broadly classified into synergistic interactions—where one compound enhances the perception of another—and antagonistic interactions, in which one compound suppresses the perception of another [12]. Among the latter, the phenomenon in which the presence of one compound reduces the perception of another, particularly an undesirable off-flavor, is known as a masking effect. Ferreira et al. have described masking as one subtype of perceptual aroma interactions [13]. Examples of masking have been reported in wine; notably, 2,4,6-trichloroanisole (TCA), the causative compound of cork taint, has been shown to suppress other aroma notes even at sub-threshold concentrations [14]. More recently, molecular mechanisms underlying masking have begun to be elucidated, including competitive binding of multiple odorants to the same olfactory receptor [15]. Building on these insights, the present study also focuses on the masking effects of phenolic off-flavors (4-ethylguaiacol and 4-ethylphenol) in the Hokkaido red wine cultivar Yamasachi.

## 2. Materials and Methods

### 2.1. Wine Samples and Chemicals

A total of 11 commercial wines (three Yamasachi wines, four each of Pinot Noir and Zweigelt) were collected for aroma compound and sensory analysis, and an additional six wines (two each of Yamasachi, Pinot Noir, and Zweigelt) for the masking test were collected from wineries in Hokkaido, Japan. Yamasachi wines were produced by different wineries in the same region (Tokachi) between 2019 and 2021. Pinot Noir and Zweigelt wines were produced by six wineries in different regions of Hokkaido, Japan. These wines were selected to represent commercially available products and to cover the typical variability associated with different wineries and production regions within Hokkaido. Although the vintages varied, the selected wines reflect commonly distributed styles of each variety and were considered representative for comparative analysis. The wines used for the masking test were different from those used for the aroma and sensory analyses in order to evaluate the aroma profile and the masking behavior independently. All wines were stored at 4 °C until analysis.

Vitispirane and linalool ethyl ether were provided by Takata Koryo Co., Ltd. (Amagasaki, Hyogo, Japan), 2-Undecanone, *cis*-3-hexen-1-ol, *trans*-2-hexen-1-ol were purchased from Tokyo Chemical Industry (Tokyo, Japan); 4-ethylguaiacol and 4-ethyphenol were purchased from Merck (Darmstadt, Germany); and Bois de Rose oxide was purchased from Toronto Research Chemicals (Vaughan, ON, Canada).

### 2.2. Wine Chemical Parameter Analysis

Standard chemical parameters of wine, including density, ethanol, glucose, fructose, lactic acid, malic acid, pH, total acid, total sugar, and volatile acidity were analyzed using the Oenofoss FT-IR analyzer (FOSS Analytical A/S, Hillerød, Denmark). Measurements were performed in triplicate for each wine.

Chemical analyses were performed according to the FT-IR reference procedures for wine analysis provided by FOSS Analytical. Units for each parameter were as follows: density (g/mL), ethanol (% *v*/*v*), glucose, fructose, lactic acid, malic acid, total acid, total sugar, and volatile acidity (all in g/L), and pH (dimensionless).

### 2.3. Aroma Compounds Analysis

Aroma compounds in wine were analyzed using headspace solid-phase microextraction (HS–SPME) coupled with gas chromatography–time-of-flight mass spectrometry (GC-TOFMS; Pegasus BT, LECO, St. Joseph, MI, USA) equipped with a PAL autosampler system (L-PAL3, LECO). Chromatographic separation was performed on an InterCap Pure-Wax capillary column (polyethylene glycol; 60 m × 0.25 mm × 0.25 μm; GL Science, Tokyo, Japan), with helium as the carrier gas at a constant flow of 1.0 mL/min.

Before extraction, 2-undecanone was added to the wine samples as an internal standard. Methyl ketones are commonly present in essential oils, foods, and dairy products, and 2-undecanone was selected because its retention time is close to those of the target aroma compounds while avoiding peak overlap. For each analysis, 8 mL of wine was transferred into a 10-mL headspace vial containing 1 g of sodium chloride, and the vial was incubated at 45 °C for 5 min.

Volatile compounds were extracted using a 50/30 μm DVB/CAR/PDMS SPME fiber (Merck Life Science, Schnelldorf, Germany). The fiber was exposed to the headspace for 30 min at 45 °C with agitation at 250 rpm. After extraction, the fiber was inserted into the GC injection port and thermally desorbed at 260 °C for 2 min in split mode (5:1). Wine samples were introduced into the GC exclusively by thermal desorption of the SPME fiber. Two HS–SPME replicates were analyzed for each wine sample.

The GC oven program was set as follows: initial temperature 40 °C for 1 min, increased to 250 °C at 10 °C/min, and held for 8 min, resulting in a total run time of 19 min. Mass spectra were acquired in full-scan mode (*m*/*z* 35–500) using electron ionization at 70 eV, with an acquisition delay of 120 s and an acquisition rate of 150 spectra/s.

The GC–TOFMS data were processed using the Chroma TOF Tile ver. 5.54 (LECO) that includes Non Target Deconvolution^®^ as part of the automated peak finding [16]. The peak areas were determined by integrating a single *m*/*z* value per analyte. All chromatogram peaks were compared with those stored at the National Institute of Standards and Technology (NIST).

### 2.4. Sensory Analysis

For the wine sensory evaluation, six expert judges (two females and four males, aged between 40 s and 60 s) were selected [17]. Individuals, including sommeliers, winemakers, and wine professionals, were recruited. Each judge has at least 20 years of experience in the wine industry. Wine samples were stored at 5 °C and served at room temperature (approx. 22 °C) in standard (INAO) wine-tasting glasses. The samples were labeled with random numbers on both the wine bottle and glass plate. Initially, the judges discussed and reached a consensus on the sensory attributes and the corresponding reference standards for wine. During panel training, reference wines were used to establish a shared understanding of the intensity range (scale perception) for each descriptor. A blinded sensory test was performed on each sample. Each taster was served 20 mL wine, who sniffed and tested the wine to detect its aroma and flavor. Nine aroma descriptors (green pepper, horseradish, earthy, mushroom, black fruit, red fruit, herbs, spices, and flowers) were selected. The tasters were asked to score the intensity of each descriptor on a scale of 0 to 10. The intensity scale was a discrete 0–10 category scale anchored at “0 = none” and “10 = very intense”. Additional descriptors mentioned by panelists were recorded qualitatively; however, because they were not consistently used across all judges, they were not included in the statistical analysis and were considered only for supplementary interpretation. In addition, the taster could describe additional sensory attributes found in each wine. They could rinse their mouths with water provided.

### 2.5. Quantitative Analysis

Quantitative analysis of the main aroma compounds (linalool ethyl ether, vitispirane, 4-ethylphenol, 4-ethylguaiacol, Bois de Rose oxide, trans-2-hexen-1-ol, and cis-3-hexen-1-ol) and the volatile phenols used in the masking tests (4-ethylphenol and 4-ethylguaiacol) was performed using GC-TOFMS. Standard aroma compounds listed in Section 2.1 were diluted in acetone to obtain a stock solution of 100 ng/L, which was further diluted to prepare five calibration levels ranging from 0.01 to 100 ng/L. A 1-mL aliquot of each calibration solution was transferred into a 20-mL headspace vial, and each level was prepared in duplicate. Calibration curves were constructed using the internal standard method with 2-undecanone to compensate for variability during sample preparation and injection.

All calibration curves showed excellent linearity across the tested range, with coefficients of determination (R^2^) greater than 0.999 for all compounds. Limits of detection (LOD) and limits of quantification (LOQ) were determined based on signal-to-noise ratios of 3 and 10, respectively. The LOD values were ≤0.1 ng/L (ppb), and the LOQ values were within the lower end of the calibration range. Recovery was evaluated by spiking wine samples with known concentrations of the target compounds, resulting in recoveries between 92% and 105%. Calibration solutions and wine samples were also used to assess method sensitivity, repeatability, and quantitative performance stability.

For HS–SPME extraction, 8 mL of wine was introduced into a 10-mL headspace vial, incubated at 30 °C for 1 min, and extracted for 30 min at the same temperature. GC oven conditions, carrier gas flow, and MS acquisition parameters were identical to those described in Section 2.3.

### 2.6. 4-Ethylguaiacol and 4-Ethylphenol Masking Test

For the masking test, six types of wines were prepared: two each of Yamasachi, Pinot Noir, and Zweigelt. These wines were selected based on their distinct aromatic characteristics. 4-ethylguaiacol was added at 30 μg/L (threshold) and 200 μg/L (average amount quantified in Yamasachi), and 4-ethylphenol was added to 420 μg/L (threshold) and 800 μg/L (double of the threshold) final concentration. These represent additional concentrations; therefore, the final concentration of each phenolic compound exceeded these values. Phenolic-additive-free wine was also prepared. In total, 30 wine samples were evaluated for their 4-ethylguaiacol and 4-ethylphenol aromas. The masking test was conducted using the same method as that used for sensory analysis. The wines were rated for phenolic aroma by six sensory experts on a 5-point scale (1: odorless, 2: slightly noticeable, 3: noticeable, 4: distinct, 5: unpleasant).

### 2.7. Statistical Analysis

Statistical analyses were performed using the JMP software Student Edition 19 (SAS Institute, Inc., Cary, NC, USA) [18]. The aroma compound data were analyzed using analysis of variance (ANOVA) to investigate the impact of different wine grape varieties. Factors that significantly contributed (*p* < 0.05) to the variation in the dataset among the three types of wine grapes were assessed for all aroma compounds. Significant chemical and sensory data were analyzed using multiple pairwise comparisons and PCA.

Sensory scores obtained in the masking test (five-point scale) were analyzed using one-way ANOVA followed by the Student–Newman–Keuls (SNK) multiple comparison test. Because the 1–5 scale is ordinal, the use of ANOVA and SNK assumes continuous data, and this methodological limitation is acknowledged in the Discussion. The assumptions of ANOVA (normality and homogeneity of variances) were evaluated using JMP diagnostic tools and were confirmed to be met. For PCA, we clarified that the data were mean-centered prior to analysis, and that PCA was performed on the full dataset of significant chemical and sensory variables.

## 3. Results

### 3.1. Chemical Parameters of Wine

Table 1 shows the results of chemical parameters of each wine. Among the general parameters, the Student–Newman–Keuls test was applied to discriminate between the means of the chemical data. Yamasachi wines had higher levels of glucose, fructose, total acid, and total sugar than the other wines. The density and pH levels were similar among all wines. The lactic acid concentrations in Yamasachi and Pinot Noir were similar, whereas Zweigelt exhibited a tendency toward lower values. Additionally, malic acid levels were lower in Pinot Noir, whereas Yamasachi and Zweigelt had nearly identical values. Zweigelt had a lower volatile acidity than the other two varieties. Although basic chemical parameters such as alcohol concentration, pH, and total acidity were measured to provide background information on the wine matrices, these variables were not incorporated into the statistical model because the objective of this study was to evaluate aroma compounds, sensory attributes, and masking behavior. In general, differences in sugars, acids, and ethanol can influence the release or perception of volatile compounds, as suggested in the previous literature; however, establishing such relationships was beyond the scope of the present work. This contextual explanation has been added to clarify the role of Table 1.

### 3.2. Identification of the Volatile Compounds in Yamasachi Wine Using GC–TOFMS

**Figure 1:**
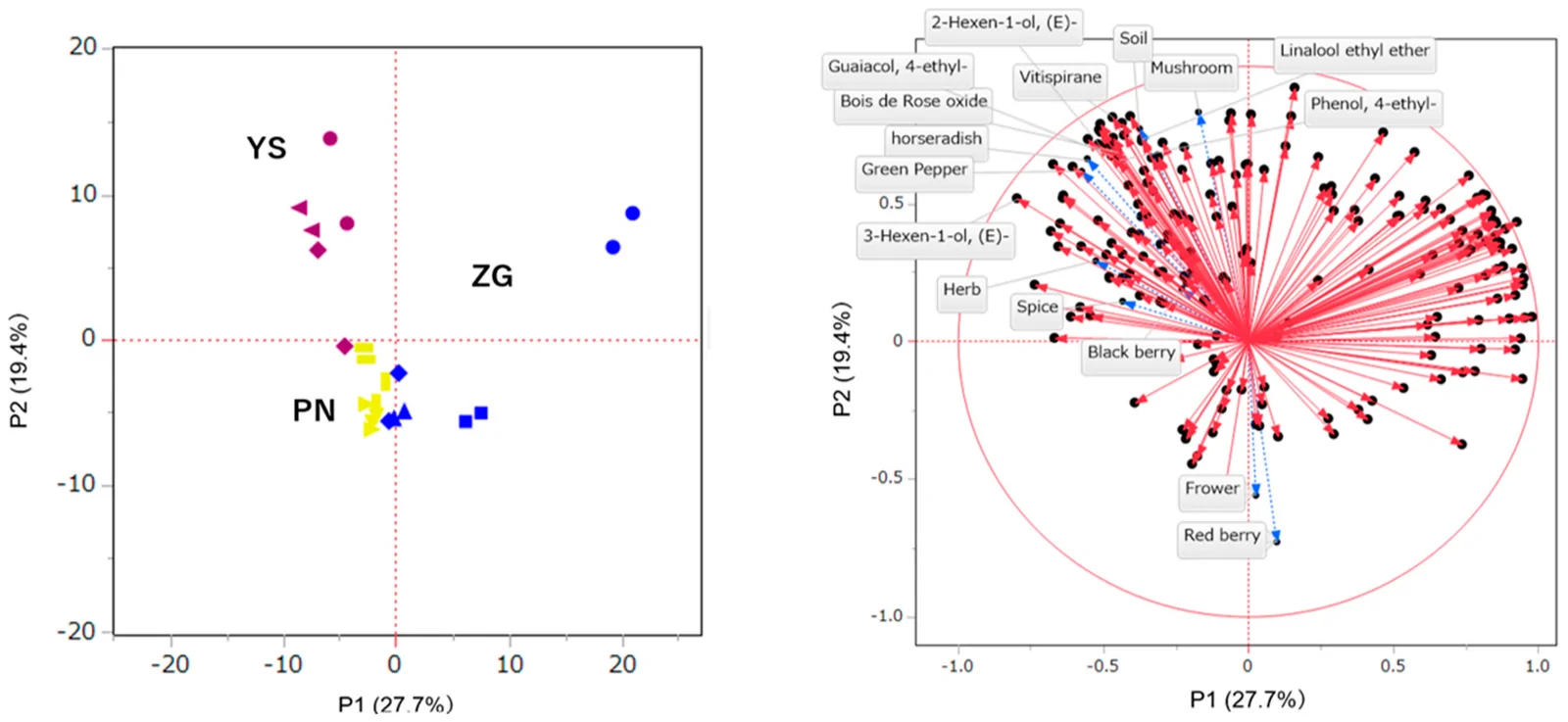
Principal component analysis (PCA) score plot and loading plot illustrating the distribution of volatile compounds and sensory attributes in Yamasachi, Pinot Noir, and Zweigelt wines. The score plot demonstrates the separation among the three wine varieties along the first two principal components, reflecting differences in their volatile profiles and sensory characteristics. Yamasachi wines are positioned distinctly from Pinot Noir and Zweigelt, indicating unique aromatic attributes. The loading plot highlights the contribution of individual volatile compounds and sensory descriptors to the observed variation.

Approximately 700 compounds were detected by non-target analysis using GC–TOFMS (Table S1). From these 700 compounds, 200 aroma compounds were extracted based on significant concentration differences between each wine. PCA was performed using the area values of the GC–TOFMS data of each compound to identify the Yamasachi-specific compounds. Based on their PCA loading values, 90 volatile compounds were selected as distinctive aroma compounds of Yamasachi, such as esters, terpenoids, norisoprenoids, alcohols, ethers, aldehydes, and phenols (Table S2). Additionally, it is worth noting that most of these compounds were tentatively identified based on mass spectral library comparisons. From these compounds, seven compounds (linalool ethyl ether, vitispirane, 4-ethyl-phenol, 4-ethyl-guaiacol, Bois de Rose oxide, *trans*-2-hexen-1-ol, and *cis*-3-hexen-1-ol) with higher area values than those in Pinot Noir and Zweigelt were identified as characteristic aromatic compounds of Yamasachi (Figure 1). The first two principal components explained 27.7% (PC1) and 19.4% (PC2) of the total variance, respectively, and were retained based on the Kaiser criterion (eigenvalues > 1). The PCA score plot showed a clear separation of Yamasachi from Pinot Noir and Zweigelt along PC1, indicating distinct differences in their volatile profiles. The loading plot revealed that compounds such as linalool ethyl ether, vitispirane, 4-ethyl-phenol, 4-ethyl-guaiacol, Bois de Rose oxide, trans-2-hexen-1-ol, and cis-3-hexen-1-ol contributed strongly to the positioning of Yamasachi wines. These compounds were located in the same direction as sensory descriptors characteristic of Yamasachi, including green pepper, earthy, mushroom, herbs, and black fruits, suggesting a chemical basis for these sensory attributes. Furthermore, several of these compounds, particularly 4-ethyl-phenol and 4-ethyl-guaiacol, have been reported to modulate aroma perception and may play a role in the masking effect observed in Yamasachi wines. This interpretation provides a clearer connection between the PCA results, the sensory profile, and the objectives of the present study.

### 3.3. Sensory Analysis

The results of the aroma analysis of the 11 wines are shown in Table 2. The Student–Newman–Keuls test was used to discriminate between the means of the sensory data. The results indicated that both Yamasachi and Zweigelt exhibited black fruit aromas, whereas the red fruits were characteristic of Pinot Noir. Floral notes were significantly higher in the Pinot Noir variety, whereas mushroom and spice aromas were detected in all varieties. Pinot Noir had the lowest herbs aroma intensity, followed by Zweigelt, with Yamasachi displaying the highest intensity. Notably, Yamasachi contributed more prominently to the perception of green pepper, earthy, and horseradish than other varieties (Figure 1).

### 3.4. Correlation of Quantitative Sensory Analysis and Key Aroma Compounds

To identify the relationship between aroma compounds and sensory analysis data (Table 2), additional PCA was performed by integrating the aroma compound profile and sensory data (Figure 1). These results indicate a unique sensory description of Yamasachi wines. Yamasachi wines are indicated on the upper left side of the figure. In contrast, the Pinot Noir and Zweigelt wines were below the middle line. The distinctive aroma and sensory characteristics of wine samples were plotted. For example, Pinot Noir and Zweigelt wines have floral and red fruit-like aromas. In contrast, the aroma attributes of Yamasachi wines are green pepper, horseradish, earthy, mushroom, black fruits, herbs, and spices. The seven volatile compounds with higher area values in GC–TOF analyses were plotted with Yamasachi wines: linalool ethyl ether, vitispirane, 4-ethylphenol, 4-ethyguaiacol, Bois de Rose oxide, *trans*-2-hexen-1-ol, and *cis*-3-hexen-1-ol, and have an aroma, as listed in Table 3. The aroma compounds and sensory characteristics of Yamasachi wines were similar (Table 3).

### 3.5. Quantitative Analysis

**Figure 2:**
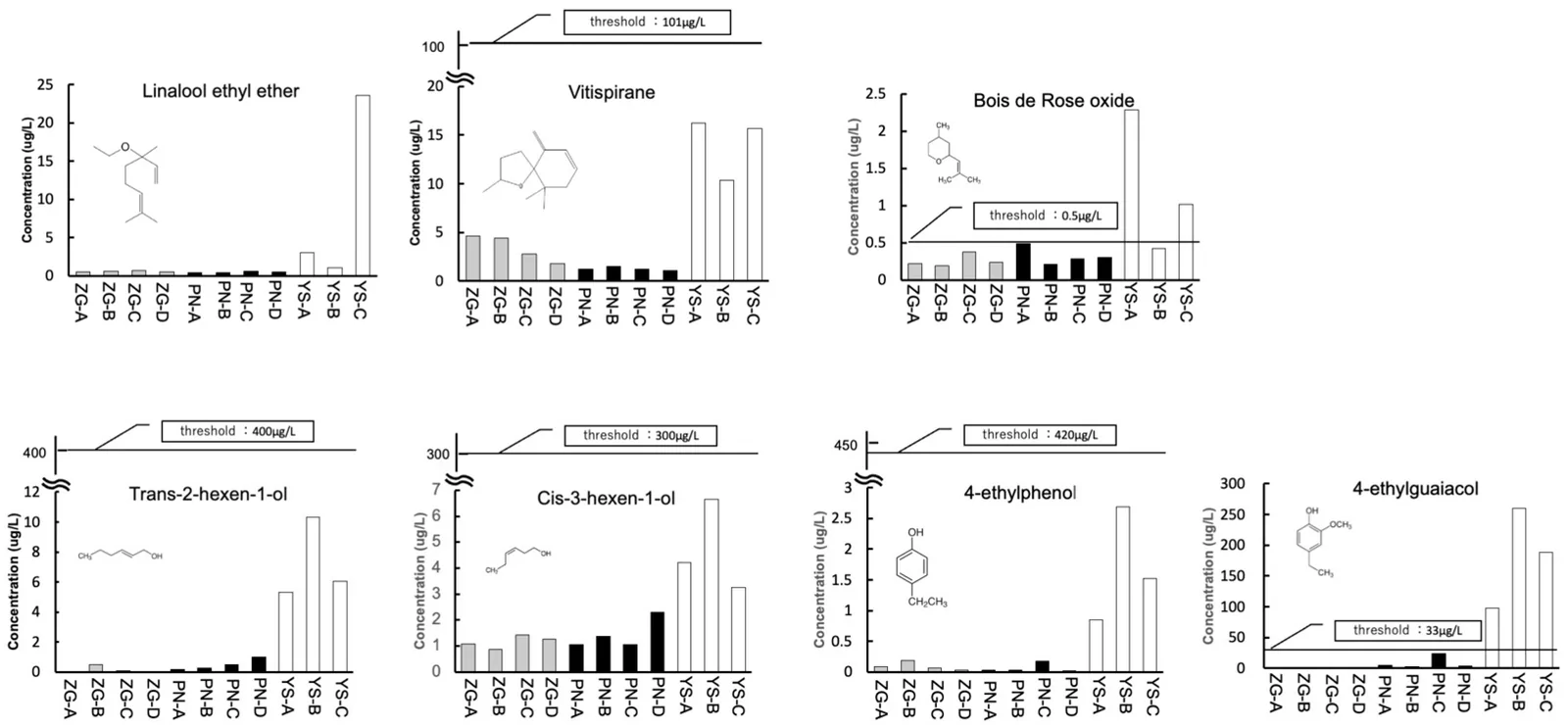
Results of the quantitative analysis of seven characteristic aroma attributes identified in Yamasachi wines through aroma compound profiling. The figure illustrates the relative concentrations of key volatile compounds. Seven representative descriptors were selected to highlight the unique aromatic signature of Yamasachi compared with those of Pinot Noir and Zweigelt. Quantitative values demonstrate that Yamasachi exhibits higher levels of these characteristic aromas than the other varieties, providing a comprehensive view of the chemical basis underlying its distinctive flavor profile.

To evaluate the effect on wine aroma, seven characteristic aroma compounds were quantified for all wine samples using standard compounds (Figure 2). vitispirane, *trans*-2-hexen-1-ol and *cis*-3-hexen-1-ol concentrations in Yamasachi wines were approximately 2–10 times higher than those in other varieties. Linalool ethyl ether concentration was approximately 2–25 times higher than those in the other varieties. The concentration of bois de rose oxide in Yamasachi wines was higher than that in other wines, except for one Yamasachi wine, which was similar to that in Pinot Noir. The concentrations of the phenolic compounds 4-ethylguaiacol and 4-ethylphenol in Yamasachi wines were significantly higher than those in the other wines: 10–35 times (4-ethylphenol) and 100–250 times (4-ethylguaiacol), confirming the results of the PCA.

In addition, quantification showed that the concentrations of two compounds, bois de rose oxide in two Yamasachi wines and 4-ethylphenol in all Yamasachi wines, were higher than their threshold concentrations (Figure 2) [19]. Particularly, 4-ethylphenol is known for “phenolic” off-flavor, and its concentration in Yamasachi wines was much higher than the threshold. In addition, although the concentration was less than the threshold, another phenolic compound, 4-ethylguaiacol, was present in all Yamasachi wines at much higher concentrations than in the other wines. However, the sensory test results showed no unpleasant smell in Yamasachi. This suggests that some compounds in Yamasachi wines may have a masking effect on phenolic off flavors. Scents play a crucial role in not only enhancing or adding aroma but also masking undesirable and unpleasant odors with another scent [20]. In addition, certain components can mask unpleasant odors [21]; therefore, the masking effect of Yamasachi wines on phenolic attributes was further tested.

### 3.6. 4-Ethylguaiacol and 4-Ethylphenol Masking Tests

An additional sensory test was designed to evaluate the masking effect of the phenolic aroma in Yamasachi wine. In the test, sensory panels evaluated the phenolic attributes of the wines after adding a certain amount of phenolic compounds. If the panelists cannot detect phenolic attributes in Yamasachi wines, whereas they can clearly perceive them in other wines containing the same amount of added phenolic compounds, this suggests that Yamasachi exhibits a masking effect on phenolic aromas.

**Figure 3:**
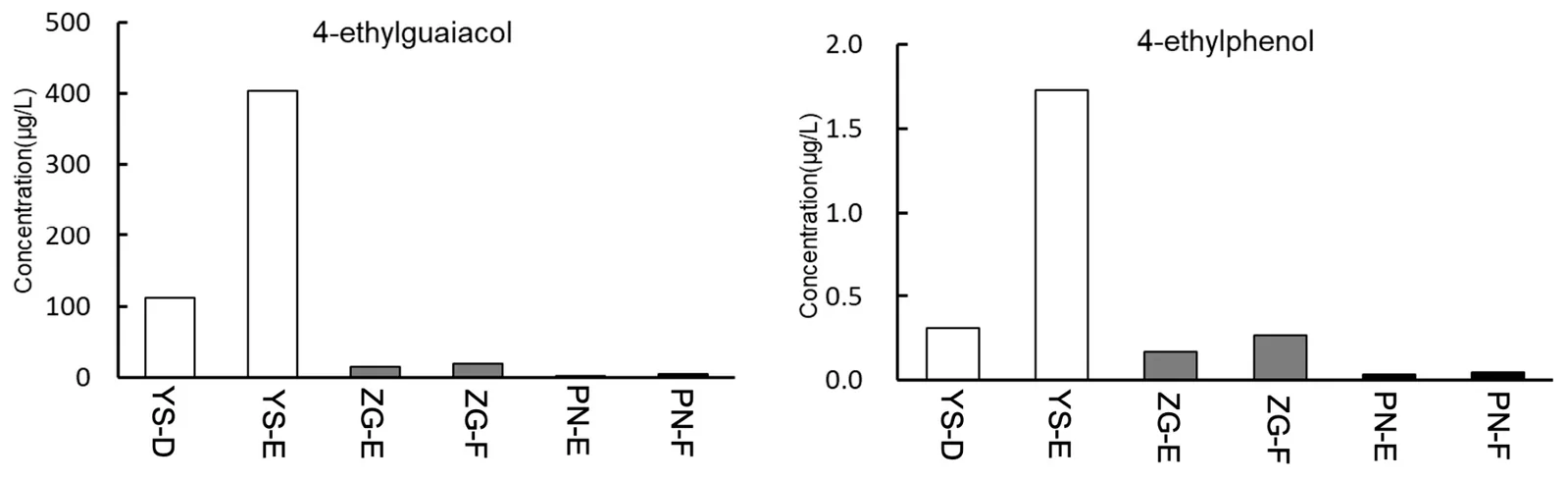
Quantitative analysis of 4-ethylguaiacol and 4-ethylphenol in six wines selected for the masking test based on aroma compound profiling. The figure confirms the levels of these phenolic compounds in the wines used, providing a basis for evaluating their contribution to sensory masking effects. Variety and vintage of used wines are: YS-D, Yamasachi 2019; YS-E, Yamasachi 2020; ZG-E, Zweigelt 2021; ZG-F, Zweigelt 2019; PN-E, Pinot Noir 2020; PN-F, Pinot Noir 2019.

First, the 4-ethylguaiacol and 4-ethylphenol concentrations in the test wines were determined (Figure 3). The 4-ethylguaiacol and 4-ethylphenol concentrations in the Yamasachi wines were higher than those in other wines. Then, each phenolic compounds were added at two different concentrations into each wine: 30 μg/L and 200 μg/L for 4-ethylguaiacol, and 420 μg/L and 800 μg/L for 4-ethylphenol. These represent additional concentration; therefore, the final concentration of each compound in a wine is more than the added concentration. For example, the final concentration of 4-ethylguaiacol in Yamasachi 2020 wines with addition of 30 μg/L, will be approximately 130 μg/L. Phenolic attributes were evaluated on a 5-point scale.

**Figure 4:**
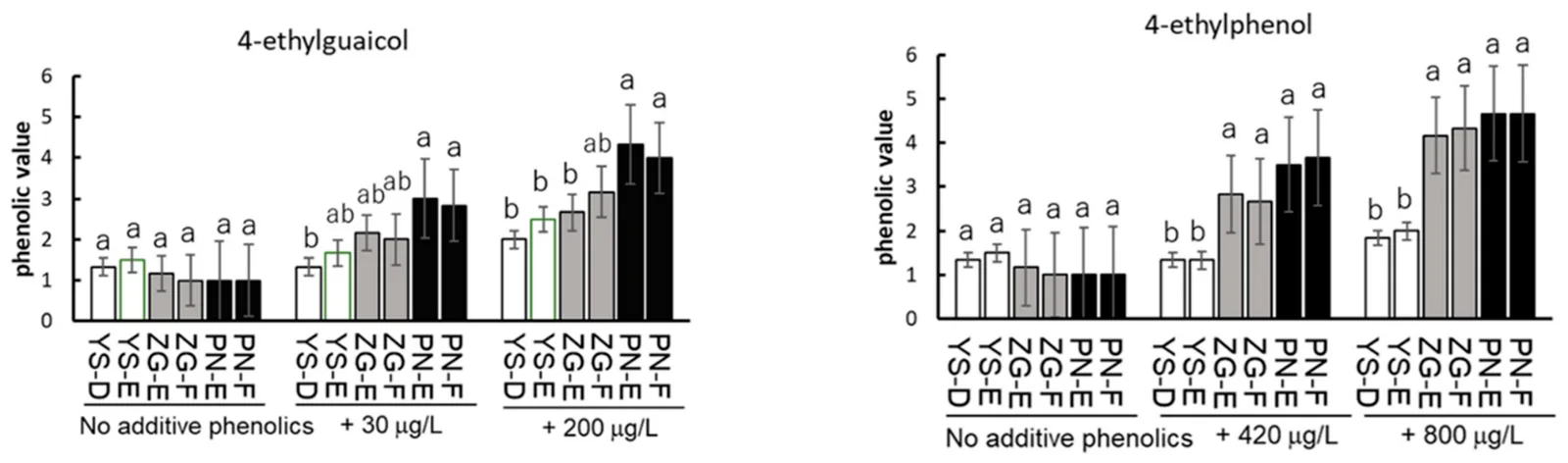
Phenol scores according to the amounts of 4-ethylguaiacol and 4-ethylphenol added to six wines (two Yamasachi, two Pinot Noir, and two Zweigelt) selected for the masking test. 4-ethylguaiacol concentrations were adjusted to 30 μg/L (threshold) and 200 μg/L (average level quantified in Yamasachi), while 4-ethylphenol was added at 420 μg/L (threshold) and 800 μg/L (twice the threshold). Sensory evaluation was conducted by six expert panelists using a five-point scale (1: odorless, 2: slightly noticeable odor, 3: noticeable odor, 4: distinct odor, 5: unpleasant odor) to assess the masking effects of these phenolic compounds. ^a,b,ab^ According to the result of the Student–Newman–Keuls test, values that do not share a common superscript are significantly different (*p* < 0.05).

The results of the masking tests are presented in Figure 4. The scored for no additive phenolics groups were low (1.0–1.5 points in all wines). When 30 μg/L 4-ethylguaiacol was added, the scores for Yamasachi wines were low (1.3–1.7 points) but increased for Pinot Noir wines (2.8–3.0 points) and Zweigelt wines (2.0–2.2 points). When 200 μg/L 4- ethylguaiacol was added, the score increased for Pinot Noir and Zweigelt (4.0–4.3 and 2.7–3.2 points, respectively) but remained low for Yamasachi wines (2.0–2.5 points), though not significant. In case of 4-ethylphenol, the masking effect was significant. Although the suppression of 4-ethylguaiacol did not reach statistical significance, the sensory scores for Yamasachi remained consistently lower than those for Pinot Noir and Zweigelt at both spike concentrations. This pattern represents a qualitative, consistent association with reduced perception of 4-ethylguaiacol, indicating a practical masking tendency despite the absence of statistical significance. After adding 420 μg/L 4-ethylphenol, the Yamasachi, Pinot Noir, and Zweigelt wines scored 1.3, 3.5–3.7, and 2.7–2.8 points, respectively. When increasing the spike concentration to 800 μg/L, the Yamasachi, Pinot Noir, and Zweigelt wines scored 1.8–2.0, 4.7, and 4.2–4.3 points, respectively. Although Yamasachi naturally contains a large amount of phenolic compounds, the masking test results indicated that the phenolic aroma was not as strongly perceived, even with the addition of extra phenolic compounds. Based on these results, a masking effect on phenolic aroma was identified in Yamasachi wines.

## 4. Discussion

In this study, seven volatile compounds co-localized with Yamasachi wines in the PCA plot: linalool ethyl ether, vitispirane, 4-ethylphenol, 4-ethylguaiacol, Bois de Rose oxide, trans-2-hexen-1-ol, and cis-3-hexen-1-ol. These compounds contribute to the aromatic profile of Yamasachi and are found in various *Vitis vinifera* cultivars. Linalool ethyl ether has been detected in Muscat-type and Gewürztraminer cultivars, where it contributes to floral and citrus-like aromas [22]. Its presence in Rieslings has also been linked to cold-climate monoterpene expression. Vitispirane is a norisoprenoid commonly found in aged red wines, such as Tempranillo, where it enhances spiciness and woody notes. Its formation via carotenoid degradation is influenced by genetic variation and aging conditions [23,24]. Bois de rose oxide is responsible for the lychee-like aroma of certain Gewürztraminer wines [25]. *trans*-2-hexen-1-ol and *cis*-3-hexen-1-ol are commonly found in white wine [26] and contribute to the green and herbaceous notes. Among these five aromatic compounds, four, except vitispirane, are traditionally associated with white wines [27], but were found at relatively high concentrations in the red wines produced from Yamasachi. Their prominence in Yamasachi red wine is noteworthy and suggests varietal-specific biosynthetic or matrix-related effects. This unusual presence may reflect a genetic lineage, cool-climate adaptation, or the influence of non-volatile components on aroma retention and release. Further research is warranted to clarify whether such patterns are common among cold-hardy hybrid cultivars or are unique to Yamasachi.

The PCA results also revealed that Yamasachi wines exhibited a distinct sensory profile characterized by green pepper, horseradish, earthy, mushroom, black fruit, herbs, and spice aromas. Notably, despite the high concentrations of 4-ethylphenol and 4-ethylguaiacol, compounds typically associated with phenolic off-flavors, these descriptors were not detected in sensory evaluations. This discrepancy strongly suggests a masking effect [28]. However, it should be noted that these mechanisms were not directly investigated in the present study. Therefore, the discussion above should be interpreted as a hypothesis for future research rather than a conclusion supported by the current experimental data.

Among the seven Yamasachi-specific volatile compounds, linalool derivatives and vitispirane are known for their floral and woody notes, which may override or suppress the smoky and medicinal characteristics of phenolic volatiles. Additionally, the presence of camphor-like, spicy, and earthy aromas may form a complex matrix that dilutes or masks phenolic perceptions. Another plausible masking candidate is the pigment composition of Yamasachi wine. Pigments such as anthocyanins and polymeric phenols, which are abundant in *Vitis amurensis*-derived cultivars, may interact with volatile phenols through non-covalent binding or co-pigmentation, possibly reducing their volatility or altering their sensory impact [29]. This hypothesis is supported by the high phenolic diversity reported in *V. amurensis* grapes, which includes unique flavonoids and stilbenes that are not commonly found in *V. vinifera*; these compounds may also contribute to visual cues that influence aroma perception [30].

Yamasachi is a hybrid of Seibel13053 and *V. amurensis*. Although Seibel hybrids are known for their disease resistance and moderate phenolic contents, they do not typically exhibit elevated volatile phenol levels. Therefore, the elevated 4-ethylphenol and 4-ethylguaiacol levels in Yamasachi wines may have originated from *V. amurensis*. Unfortunately, the parental tree of *V. amurensis* used in the Yamasachi breeding has been lost. Therefore, clarifying the genetic origin of the elevated phenolic compounds is difficult, but the reason could be uncovered using biochemical approaches, such as phenolic compound quantification in Yamasachi fruits and/or vines.

The ability to mask phenolic off-flavors has practical implications for winemaking, particularly in cool climates or hybrid grape regions where *Brettanomyces*-like aromas are more prevalent [31]. Understanding the phenolic off-flavor masking mechanisms in Yamasachi wines could inform blending strategies, yeast selection, and targeted breeding for sensory resilience. Future research should focus on identifying compounds responsible for masking, interaction mechanisms, and thresholds in complex wine matrices [32].

This study has several limitations that arise primarily from the restricted availability of Yamasachi wines and the methodological scope of the sensory experiments. First, the limited number of samples—reflecting the low production of this regional variety—prevented the inclusion of multiple vintages and regions, and the masking test relied on only two samples per variety, resulting in pseudo-replicates and reduced statistical power. Second, the sensory evaluation depended solely on trained panelists and used an ordinal 1–5 scale, and repeatability assessments were not conducted; these methodological constraints may influence the generalizability and robustness of the sensory results. Third, although the masking mechanism was inferred from existing literature, targeted verification through pigment or polyphenol addition tests or GC-Olfactometry was beyond the scope of this study, and chemical parameters such as alcohol concentration, pH, and total acidity were not incorporated into the statistical model.

Despite these constraints, this work represents the first systematic characterization of the volatile and sensory profile of Yamasachi using non-target GC-TOFMS combined with sensory evaluation and multivariate analysis. The seven characteristic compounds identified here collectively form an aroma fingerprint of the variety and provide a valuable reference for future research on hybrid grape cultivars in cool-climate regions. The masking interactions observed in Yamasachi wines may also inform broader strategies for off-flavor improvement in other wines and food products.

Future studies should expand the dataset to include additional vintages, regions, and other *V. amurensis*–derived cultivars [33,34], incorporate consumer panels and repeated sensory evaluations, and apply descriptive methods such as sensory wheels or CATA. Mechanistic verification through additive-based sensory tests and GC-Olfactometry, along with chemometric modeling approaches such as PLS or random forests, will further clarify the contribution of phenolic compounds to aroma perception. Investigating the influence of fermentation conditions, yeast strains, and oak aging on the formation of characteristic compounds will also provide practical insights for winemaking control.

## 5. Conclusions

In this study, seven key aroma compounds—linalool ethyl ether, Bois de rose oxide, vitispirane, *trans*-2-hexen-1-ol, *cis*-3-hexen-1-ol, 4-ethylguaiacol, and 4-ethylphenol—and their sensory attributes were identified in Yamasachi wine. Quantitative analysis revealed that the off-flavor compounds 4-ethylguaiacol and 4-ethylphenol were more abundant in Yamasachi wine than in other wines. The masking tests demonstrated a statistically significant suppression of 4-ethylphenol, whereas the effect on 4-ethylguaiacol did not reach statistical significance. Nevertheless, Yamasachi consistently showed much lower sensory scores for 4-ethylguaiacol than Pinot Noir and Zweigelt at the same spike concentrations, indicating a practical masking tendency relative to these cultivars. Therefore, the masking behavior of Yamasachi should be interpreted as compound-specific rather than a general suppression of all phenolic aromas. Taken together, these findings suggest that, although statistical significance was not reached for 4-ethylguaiacol, Yamasachi exhibits a consistent qualitative association with reduced perception of this compound, supporting a practical masking tendency in line with the sensory results. Although the precise mechanism underlying this masking effect remains to be experimentally verified in future studies, these findings demonstrate the uniqueness of Yamasachi grapes and suggest that Yamasachi wine may offer a new approach to suppressing phenolic off-flavors.

## Figures and Tables

**Table 1:** Chemical parameters of wines used in this study.

	YS-A	YS-B	YS-C	PN-A	PN-B	PN-C	PN-D	ZG-A	ZG-B	ZG-C	ZG-D
Variety	Yamasachi	Yamasachi	Yamasachi	Pinot Noir	Pinot Noir	Pinot Noir	Pinot Noir	Zweigelt	Zweigelt	Zweigelt	Zweigelt
Vintage	2019	2020	2021	2017	2020	2019	2020	2019	2020	2016	-
Density (g/mL)	0.9940 ^b^ (0.0005)	0.9948 ^b^ (0.0002)	0.0.9978 ^a^ (0.0002)	0.9930 ^cd^ (0.0005)	0.9926 ^cde^ (0.0003)	0.9920 ^e^ (0.0003)	0.9942 ^b^ (0.0003)	0.9945 ^b^ (0.0003)	0.9923 ^de^ (0.0003)	0.9932 ^c^ (0.0005)	0.9927 ^cde^ (0.0006)
Ethanol (% Vol)	11.93 ^ab^ (0.35)	10.13 ^c^ (0.21)	9.80 ^c^ (0.17)	12.47 ^a^ (0.35)	11.30 ^b^ (0.35)	11.90 ^ab^ (0.44)	11.50 ^ab^ (0.46)	11.93 ^ab^ (0.25)	12.07 ^ab^ (0.42)	12.27 ^ab^ (0.35)	11.60 ^ab^ (0.46)
Glucose (g/L)	2.23 ^b^ (0.06)	1.73 ^c^ (0.12)	2.83 ^a^ (0.06)	2.33 ^b^ (0.15)	0.77 ^d^ (0.12)	0.50 ^e^ (0.00)	0.30 ^f^ (0.10)	1.73 ^c^ (0.06)	0.53 ^e^ (0.06)	0.27 ^f^ (0.12)	0.33 ^f^ (0.06)
Fructose (g/L)	0.33 ^bcd^ (0.06)	0.43 b ^c^ (0.06)	0.57 ^b^ (0.06)	0.17 ^de^ (0.06)	0.27 ^cd^ (0.15)	0.33 ^bcd^ (0.12)	0.33 ^bcd^ (0.06)	0.77 ^a^ (0.06)	0.17 ^de^ (0.12)	0.00 ^e^ (0.00)	0.37 ^bcd^ (0.15)
Lactic Acid (g/L)	2.70 ^d^ (0.17)	3.13 ^c^ (0.06)	4.60 ^a^ (0.10)	2.93 ^c^ (0.15)	3.00 ^c^ (0.10)	2.27 ^e^ (0.15)	3.57 ^b^ (0.12)	1.30 ^g^ (0.00)	1.30 ^g^ (0.00)	1.87 ^f^ (0.06)	1.47 ^g^ (0.06)
Malic Acid (g/L)	0.47 ^b^ (0.06)	0.33 ^bcd^ (0.06)	0.40 ^bc^ (0.00)	0.00 ^e^ (0.00)	0.07 ^e^ (0.06)	0.30 ^cd^ (0.10)	0.23 ^d^ (0.06)	0.43 ^bc^ (0.06)	0.43 ^bc^ (0.06)	0.23 ^d^ (0.06)	0.63 ^a^ (0.06)
pH	3.36 ^k^ (0.02)	3.50 ^f^ (0.01)	3.64 ^b^ (0.01)	3.40 ^j^ (0.01)	3.44 ^i^ (0.00)	3.54 ^d^ (0.01)	3.70 ^a^ (0.01)	3.55 ^c^ (0.00)	3.49 ^g^ (0.01)	3.47 ^h^ (0.00)	3.52 ^e^ (0.01)
Total Acid (g/L)	4.47 ^b^ (0.21)	4.03 ^c^ (0.06)	5.00 ^a^ (0.10)	3.57 ^d^ (0.12)	3.77 ^d^ (0.15)	2.93 ^f^ (0.12)	3.37 ^e^ (0.12)	3.30 ^e^ (0.00)	3.03 ^f^ (0.06)	3.60 ^d^ (0.00)	2.90 ^f^ (0.00)
Total Sugar (g/L)	3.30 ^a^ (0.26)	2.43 ^b^ (0.06)	3.13 ^a^ (0.06)	3.00 ^a^ (0.26)	0.90 ^c^ (0.10)	0.77 ^c^ (0.15)	0.80 ^c^ (0.26)	3.10 ^a^ (0.10)	1.03 ^c^ (0.21)	0.73 ^c^ (0.76)	1.43 ^c^ (0.12)
Volatile Acid (g/L)	0.46 ^e^ (0.01)	0.52 ^d^ (0.01)	0.67 ^b^ (0.01)	0.51 ^d^ (0.01)	0.77 ^a^ (0.02)	0.60 ^c^ (0.02)	0.67 ^b^ (0.04)	0.39 ^f^ (0.01)	0.43 ^ef^ (0.01)	0.51 ^d^ (0.06)	0.32 ^g^ (0.02)

Mean concentrations are shown. Standard deviations are shown in parentheses (*n* = 3). ^a–k^ According to the result of the Student-Newman-Keuls test, values that do not share a common superscript are significantly different (*p* < 0.05).

**Table 2:** Aroma sensory analysis of wines.

	YS-A	YS-B	YS-C	PN-A	PN-B	PN-C	PN-D	ZG-A	ZG-B	ZG-C	ZG-D
black fruits	5.83 ^a^ (1.17)	5.17 ^a^ (1.47)	5.67 ^a^ (1.75)	3.50 ^a^ (1.52)	3.50 ^a^ (1.87)	3.67 ^a^ (2.16)	3.67 ^a^ (1.97)	3.50 ^a^ (1.87)	6.33 ^a^ (2.34)	5.50 ^a^ (2.26)	5.33 ^a^ (2.16)
red fruits	4.83 ^a^ (2.04)	5.33 ^a^ (2.16)	4.67 ^a^ (1.51)	5.50 ^a^ (2.43)	6.50 ^a^ (1.97)	5.83 ^a^ (2.48)	7.17 ^a^ (1.83)	5.33 ^a^ (1.86)	6.00 ^a^ (1.67)	5.67 ^a^ (2.42)	5.67 ^a^ (1.86)
flower	4.33 ^a^ (1.21)	4.50 ^a^ (1.88)	4.17 ^a^ (1.47)	4.00 ^a^ (1.41)	5.83 ^a^ (2.23)	6.00 ^a^ (0.89)	5.00 ^a^ (2.10)	4.33 ^a^ (1.37)	5.50 ^a^ (1.05)	3.67 ^a^ (1.86)	5.17 ^a^ (1.72)
green pepper	7.17 ^a^ (1.72)	6.33 ^a^ (2.42)	6.17 ^a^ (1.72)	2.17 ^b^ (1.83)	2.33 ^b^ (2.42)	2.50 ^b^ (1.64)	1.50 ^b^ (0.84)	1.67 ^b^ (0.82)	2.67 ^b^ (1.86)	3.50 ^b^ (2.51)	2.83 ^b^ (2.56)
earthly	7.00 ^a^ (1.90)	5.50 ^ab^ (2.17)	5.67 ^ab^ (1.86)	4.00 ^ab^ (1.79)	4.17 ^ab^ (2.48)	4.50 ^ab^ (1.52)	4.00 ^ab^ (1.26)	4.83 ^ab^ (1.83)	3.17 ^b^ (2.40)	4.50 ^ab^ (1.87)	3.67 ^ab^ (2.25)
mushroom	5.50 ^a^ (1.64)	5.00 ^a^ (1.79)	5.17 ^a^ (1.47)	4.50 ^a^ (1.52)	4.67 ^a^ (2.16)	4.50 ^a^ (1.87)	4.67 ^a^ (1.63)	5.00 ^a^ (0.89)	4.50 ^a^ (1.38)	4.33 ^a^ (1.86)	4.67 ^a^ (1.36)
horseradish	6.50 ^a^ (2.17)	5.83 ^a^ (2.93)	6.00 ^a^ (2.45)	1.50 ^b^ (0.84)	2.33 ^b^ (2.07)	1.33 ^b^ (0.52)	2.17 ^b^ (1.60)	1.67 ^b^ (1.03)	1.67 ^b^ (0.52)	2.67 ^b^ (1.97)	3.00 ^b^ (3.10)
herb	5.33 ^a^ (2.50)	6.00 ^a^ (1.10)	6.17 ^a^ (1.60)	3.83 ^a^ (1.47)	4.67 ^a^ (1.51)	4.83 ^a^ (1.60)	4.67 ^a^ (2.42)	3.67 ^a^ (0.82)	5.50 ^a^ (0.55)	4.67 ^a^ (1.03)	4.33 ^a^ (1.37)
spice	6.00 ^a^ (2.10)	7.00 ^a^ (1.55)	6.83 ^a^ (1.17)	5.33 ^a^ (1.63)	5.83 ^a^ (1.60)	6.33 ^a^ (2.07)	6.33 ^a^ (2.16)	5.67 ^a^ (1.63)	5.33 ^a^ (1.51)	6.83 ^a^ (0.41)	6.50 ^a^ (0.84)

Mean values are shown. Standard deviations are shown in parentheses (*n* = 6). ^a,b^ According to the result of the Student-Newman-Keuls test, values that do not share a common superscript are significantly different (*p* < 0.05).

**Table 3:** Aroma substances strongly association with sensory analysis of Yamasachi.

—		Aroma Descriptors
Aroma compounds of Yamasachi	Linalool ethyl ether	flower
	Vitispirane	floral, fruity, earthy, forest
	4-ethylphenol	smoking, chemicals, band-aids
	4-ethylguaiacol	spices, smoked, medicines, cloves
	Bois de Rose oxide	camphor, herbs, rosemary
	(*E*)-2-hexen-1-ol	greens, fruity, vegetables, herbs
	(*E*)-3-hexen-1-ol	greens, peel, leaf, flower, soil
sensory analysis of Yamasachi	green pepper, horseradish, soil (earthy), mushroom, black fruit, herbs, spices	

Aroma compounds strongly correlated with the characteristic sensory attributes of Yamasachi wine. Seven volatile compounds—including linalool ethyl ether, vitispirane, 4-ethylphenol, 4-ethylguaiacol, Bois de Rose oxide, (*E*)-2-hexen-1-ol, and (*E*)-3-hexen-1-ol—showed strong consistency with Yamasachi-specific aromas such as green pepper, horseradish, earthy, mushroom, black fruits, herbs, and spices.

## Data Availability

The raw data supporting the conclusions of this article will be made available by the authors on request.

## Supplementary Materials

The following supporting information can be downloaded at https://www.mdpi.com/article/10.3390/foods15183195/s1. Table S1: Full GC–TOFMS peak list for Yamasachi, Pinot Noir, and Zweigelt wines. Untargeted GC–TOFMS volatile compound profiling of Yamasachi wine. The table includes compound names, CAS numbers, molecular formulas, retention times, retention indices, quantification ions, and peak areas. Data were extracted from LECO ChromaTOF software and reformatted for clarity. Units for retention time (min) and peak area (arbitrary units) are indicated. Sample IDs correspond to individual wine replicates analyzed using HS-SPME extraction. Table S2: Result of PCA of Yamasachi wines. Principal component analysis (PCA) loadings of volatile compounds in Yamasachi wines. The table reports loading values for principal component 1 (PC1) and principal component 2 (PC2) for each compound. Volatile compounds were detected using untargeted GC–TOFMS with HS-SPME extraction. PCA loadings indicate the contribution of each compound to the variance explained by the principal components and help characterize aroma differences among wine samples.

## Abbreviations

GC–TOFMS	Gas Chromatography–Time-of-Flight Mass Spectrometry
HS–SPME	Headspace Solid-Phase Microextraction
IS	Internal Standard
*m*/*z*	Mass-to-Charge Ratio
PCA	Principal Component Analysis
ANOVA	Analysis of Variance
OIV	International Organisation of Vine and Wine
*V. amurensis*	*Vitis amurensis*
*V. vinifera*	*Vitis vinifera*
YM	Yamasachi
PN	Pinot Noir
ZW	Zweigelt

## References

[1] Horiuchi R., Arakawa K., Kasuga J., Suzuki T., Jitsuyama Y. (2021). Freezing Resistance and Behavior of Winter Buds and Canes of Wine Grapes Cultivated in Northern Japan. Cryobiology.

[2] Li M.Y., Pei X.X., Shi N., Yang Y.M., Fan S.T., Sun Y.F., Kong Q.S., Duan C.Q., Yu K., Wang J. (2024). Volatomic Differences among *Vitis amurensis* Cultivars and Its Hybrids with *V. Vinifera* Revealed the Effects of Genotype, Region, and Vintage on Grape Aroma. Food Res. Int..

[3] Kasuga J., Tsumura Y., Kondoh D., Jitsuyama Y., Horiuchi R., Arakawa K. (2020). Cryo-Scanning Electron Microscopy Reveals That Supercooling of Overwintering Buds of Freezing-Resistant Interspecific Hybrid Grape ‘Yamasachi’ Is Accompanied by Partial Dehydration. J. Plant Physiol..

[4] Sun Y., Wang M., Cao W., Ma M., Xu P., Li C., Pan Y., Lu W. (2025). Analysis of Volatile Organic Compounds in Wines from *Vitis amurensis* Varieties in Xinjiang, China. Foods.

[5] Delgado J.A., Sánchez-Palomo E., Osorio Alises M., González Viñas M.A. (2022). Chemical and Sensory Aroma Typicity of La Mancha Petit Verdot Wines. LWT.

[6] Sánchez-Palomo E., Delgado J.A., Ferrer M.A., Viñas M.A.G. (2019). The Aroma of La Mancha Chelva Wines: Chemical and Sensory Characterization. Food Res. Int..

[7] Garbay J., Cameleyre M., Le Menn N., Riquier L., Barbe J.C., Lytra G. (2024). Study of the Fruity Aroma of Red Wines Made from Grape Varieties Potentially Adapted to Climate Change Using a Semi-Preparative HPLC Method. LWT.

[8] Toulaki A., Kalompatsios D., Mantiniotou M., Athanasiadis V., Roufas K., Lalas S.I. (2026). Correlating Chemical Fingerprint to Sensory Evaluation: A Four-Vintage Study (2017–2020) of Xinomavro Red Wine. Foods.

[9] Filipe-Ribeiro L., Cosme F., Nunes F.M. (2018). Reducing the Negative Sensory Impact of Volatile Phenols in Red Wine with Different Chitosans: Effect of Structure on Efficiency. Food Chem..

[10] Sudol P.E., Galletta M., Tranchida P.Q., Zoccali M., Mondello L., Synovec R.E. (2022). Untargeted Profiling and Differentiation of Geographical Variants of Wine Samples Using Headspace Solid-Phase Microextraction Flow-Modulated Comprehensive Two-Dimensional Gas Chromatography with the Support of Tile-Based Fisher Ratio Analysis. J. Chromatogr. A.

[11] Ma T., Sam F.E., Didi D.A., Atuna R.A., Amagloh F.K., Zhang B. (2022). Contribution of Edible Flowers on the Aroma Profile of Dealcoholized Pinot Noir Rose Wine. LWT.

[12] Styger G., Prior B., Bauer F.F. (2011). Wine Flavor and Aroma. J. Ind. Microbiol. Biotechnol..

[13] Ferreira V., De-La-Fuente-Blanco A., Sáenz-Navajas M.P. (2021). A New Classification of Perceptual Interactions between Odorants to Interpret Complex Aroma Systems. Application to Model Wine Aroma. Foods.

[14] Tempere S., Schaaper M.H., Cuzange E., de Revel G., Sicard G. (2017). Masking of Several Olfactory Notes by Infra-Threshold Concentrations of 2,4,6-Trichloroanisole. Chemosens. Percept..

[15] Hu B., Zheng H., Liang H., Lv H., Xiao Q., Jiang Y., Lu Q., Niu Y., Wang H., Xiao Z. (2026). Elucidating Odorant Masking Effects in Food Matrices Through Olfactory Receptor-Based Profiling. J. Agric. Food Chem..

[16] Tang P., Wang L., Zhao Q., Lu J., Qiao M., Li C., Xiao D., Guo X. (2024). Characterization of Key Aroma Compounds and Relationship between Aroma Compounds and Sensory Attributes in Different Quality of High Temperature Daqu. LWT.

[17] Barton A., Hayward L., Richardson C.D., McSweeney M.B. (2020). Use of Different Panellists (Experienced, Trained, Consumers and Experts) and the Projective Mapping Task to Evaluate White Wine. Food Qual. Prefer..

[18] Ma Y., Xu Y., Tang K. (2024). Molecular Descriptors of Icewine Odorants: A First Insight into Their Relationship with Metabolism and Olfactory Perception. J. Food Sci..

[19] Yang W., You Y., Ling M., Ye D., Shi Y., Duan C., Lan Y. (2023). Identification of the Key Odor-Active Compounds Responsible for Varietal Smoky Aroma in Wines Made from the East Asian Species. Food Res. Int..

[20] Tao M., Guo W., Liang J., Liu Z. (2025). Unraveling the Key Cooked Off-Flavor Compounds in Thermally Sterilized Yamasa Tea Beverages, and Masking Effect of Tea Raw Material Baking. Food Chem..

[21] Chen L., Yang F., Jiang Q., Gao P., Xia W., Yu D. (2024). Effect of Different Starch on Masking Fishy Odor Compounds. Int. J. Biol. Macromol..

[22] Darnal A., Ceccon A., Magni M., Robatscher P., Poggesi S., Boselli E., Longo E. (2024). Enantioselective-GCxGC Determination of α-Terpinyl Ethyl Ether in Wine: Quantitative Analysis and Identification of Main Terpene Precursors. Appl. Food Res..

[23] Silva Ferreira A.C., Guedes De Pinho P. (2004). Nor-Isoprenoids Profile during Port Wine Ageing—Influence of Some Technological Parameters. Anal. Chim. Acta.

[24] Song J., Smart R.E., Dambergs R.G., Sparrow A.M., Wells R.B., Wang H., Qian M.C. (2014). Pinot Noir Wine Composition from Different Vine Vigour Zones Classified by Remote Imaging Technology. Food Chem..

[25] Ruiz-García L., Hellín P., Flores P., Fenoll J. (2014). Prediction of Muscat Aroma in Table Grape by Analysis of Rose Oxide. Food Chem..

[26] Slegers A., Angers P., Pedneault K. (2017). Volatile Compounds from Must and Wines from Five White Grape Varieties. J. Food Chem. Nanotechnol..

[27] González-Barreiro C., Rial-Otero R., Cancho-Grande B., Simal-Gándara J. (2015). Wine Aroma Compounds in Grapes: A Critical Review. Crit. Rev. Food Sci. Nutr..

[28] Ferreira V., López R., Cacho J.F. (2000). Quantitative Determination of the Odorants of Young Red Wines from Different Grape Varieties. J. Sci. Food Agric..

[29] Sáenz-Navajas M.P., Campo E., Culleré L., Fernández-Zurbano P., Valentin D., Ferreira V. (2010). Effects of the Nonvolatile Matrix on the Aroma Perception of Wine. J. Agric. Food Chem..

[30] Liang Z., Wu B., Fan P., Yang C., Duan W., Zheng X., Liu C., Li S. (2008). Anthocyanin Composition and Content in Grape Berry Skin in Vitis Germplasm. Food Chem..

[31] Sun X., Luo X., Ma T., You Y., Huang W., Zhan J. (2017). Detection Method Optimization, Dynamic Changes during Alcoholic Fermentation and Content Analysis of “Brett Character” Compounds 4-Ethylphenol (4-EP) and 4-Ethylguaiacol (4-EG) in Chinese Red Wines. Food Anal. Methods.

[32] Escudero A., Campo E., Fariña L., Cacho J., Ferreira V. (2007). Analytical Characterization of the Aroma of Five Premium Red Wines. Insights into the Role of Odor Families and the Concept of Fruitiness of Wines. J. Agric. Food Chem..

[33] Shu N., Lu W., Yang Y., Cao W., Wen J., Yan Y., Liu X., Wen L. (2026). Major Volatile Aroma Composition, Organic Acids, and Color Characteristics: A Comparative Study of Berries and Wines from *Vitis amurensis* and Its Interspecific Hybrids. Foods.

[34] Sternad Lemut M., Antalick G., Comuzzo P., Voce S. (2026). Traditional and Emerging Maceration Techniques in Red Winemaking: Extraction Mechanisms Shaping Phenolic Composition, Volatile Profile, and Sensory Expression. Foods.

